# Syndrome de Nicolau compliquant une injection intramusculaire de benzathine penicilline

**DOI:** 10.11604/pamj.2014.18.105.4652

**Published:** 2014-05-30

**Authors:** Sarra Benmiloud, Moustapha Hida

**Affiliations:** 1 Service de pédiatrie, Hôpital Mère-enfant, CHU Hassan II, Faculté de médecine et de pharmacie, Université Sidi Mohamed Ben Abdellah, Fès, Maroc

**Keywords:** Syndrome de nicolau, injection, benzathine penicilline, Nicolau syndrome, injection, benzathine penicillin

## Image en medicine

Il s'agit d'une fille âgée de 6 ans, suivie pour un rhumatisme articulaire aigu, mise sous benzathine-pénicilline toutes les 3 semaines, ayant présenté immédiatement après l'injection intramusculaire de benzathine-pénicilline une douleur intense de la fesse gauche, suivie 30 minutes plus tard par l'apparition d'une macule rouge violacée étendue au niveau de la fesse gauche associée à un rash cutané livédoïde au niveau du membre inférieur homolatéral. La prise en charge était basée sur un traitement antalgique (paracétamol) et l'administration sous cutané d'héparine de bas poids moléculaire. L’évolution était favorable après 10 jours. Le diagnostic retenu est celui d'un syndrome de Nicolau, ou dermite livedoïde, ou embolia cutis medicamentosa, qui est une complication rare et sévère des injections intramusculaires. Rarement, il peut compliquer une injection intra-articulaire, intraveineuse ou sous-cutanée. Son mécanisme est mal élucidé. On suggère des mécanismes vasculaires prépondérants à type de vasospasme, de thrombose artérielle et d'embolie, un traumatisme direct par l'aiguille d'injection ou une compression péri-vasculaire par le volume injecté. Cliniquement, le patient présente une douleur intense immédiatement après l'injection, suivie par l'apparition des lésions maculeuses érythémateuses puis livédoïdes violacées, avec une extension de taille variable et une possibilité de nécrose et d'ulcérations pouvant s’étendre profondément aux plans sous-jacents. L’évolution est imprévisible avec un risque d'ischémie du membre, une surinfection cutanée, une parésie ou paralysie, des douleurs neuropathiques, ou une rhabdomyolyse parfois fatale. Le traitement est symptomatique (antalgique, parage des nécroses cutanées) avec parfois le recours à l'oxygènothérapie hyperbare ou l'héparinothérapie.

**Figure 1:**
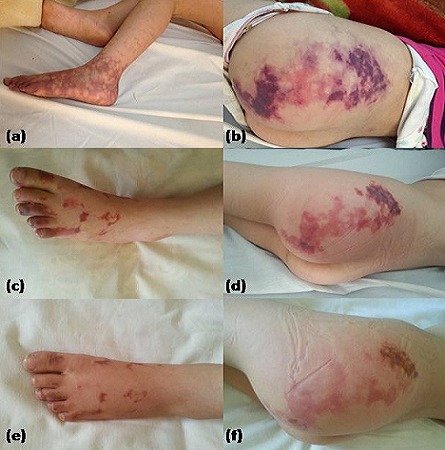
Évolution des lésions cutanées: au 1er jour (a et b) rash cutané livédoïde au niveau de la jambe et du pied gauches avec lésions ecchymotiques infiltrant la fesse gauche. Au 6ème jour (c et d) régression partielle du rash avec apparition de plages d'ischémie en cartes géographiques. Au 10ème jour (e et f) hypopigmentation et début de disparition des lésions

